# Supplementary material for: Fluorescent tools for the standardized work in Gram-negative bacteria
Source: J Biol Eng. 2024 Apr 8;18:25. doi: 10.1186/s13036-024-00420-9 (PMC11003136; doi:10.1186/s13036-024-00420-9)
Supplement: Supplementary file 1 — Supplementary Material 1. [file 13036_2024_420_MOESM1_ESM.docx]

**Supplemental information**

**Table S1: Spectra ranges for endpoint measurement determination of FPs used in this study**

| Settings for measurements | | | | | | |
| --- | --- | --- | --- | --- | --- | --- |
|  | Endpoint | | Excitation spectrum | | Emission spectrum | |
| FP | Excitation | Set  emission | Set Emission | Range | Set Excitation | Range |
| mCerulean | 452 | 482 | 515 | 300-485 | 408 | 438-700 |
| sfmTurquoise2ox | 455 | 485 | 515 | 300-485 | 408 | 438-700 |
| sfGFP | 492 | 522 | 550 | 300-520 | 460 | 490-700 |
| mGFPmut2 | 489 | 519 | 550 | 300-520 | 460 | 490-700 |
| eGFP | 494 | 524 | 550 | 300-520 | 460 | 490-700 |
| miniSOG | 453 | 522 | 541 | 300-511 | 422 | 452-700 |
| mVenusNB | 517 | 547 | 570 | 300-527 | 492 | 522-700 |
| Ypet | 517 | 547 | 570 | 300-527 | 492 | 522-700 |
| mNeonGreen | 506 | 536 | 557 | 300-527 | 481 | 511-700 |
| mRuby3 | 557 | 596 | 632 | 300-602 | 533 | 563-700 |
| mScarlet | 571 | 601 | 632 | 300-602 | 533 | 563-700 |
| mCherry | 585 | 615 | 650 | 300-620 | 564 | 594-700 |
| mCherry2-L | 583 | 613 | 650 | 300-620 | 564 | 594-700 |
| mNeptune2 | 600 | 651 | 691 | 300-661 | 574 | 604-700 |
| mNeptune2.5 | 597 | 639 | 691 | 300-661 | 574 | 604-700 |


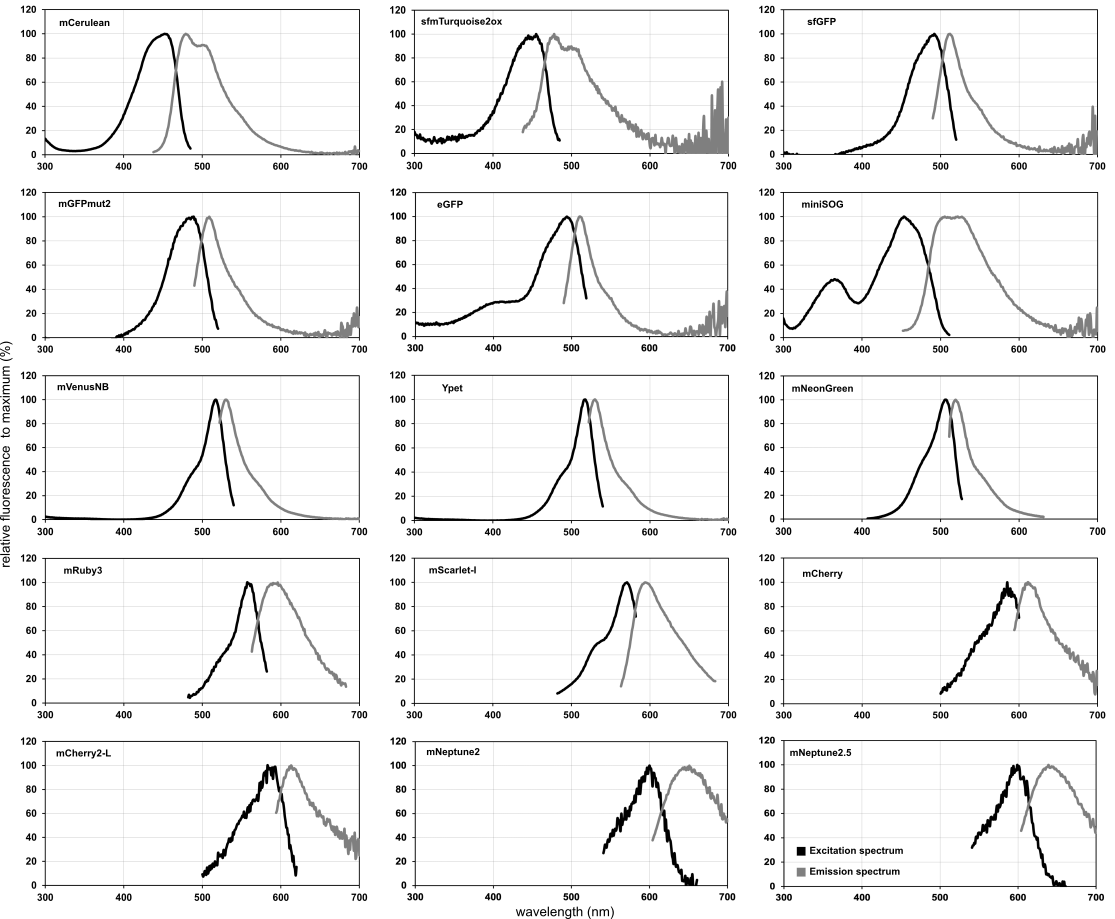


**Figure S1 Excitation and Emission spectra of FPs used in this study.**

Strains grown into exponential growth phase, harboring plasmids encoding respective FP driven by P*_rpsM_* were probed for their excitation and emission based on the nm ranges stated in table S1 using a Synergy H1 plate reader.

**Table S2 Half-life determination of FPs SsrA degradation kinetics**

| **FP** | **Tag** | **T_25_ (min)** | **T_50_ (min)** |
| --- | --- | --- | --- |
| mCerulean | LVA | 29.4 | 55.2 |
|  | AAV | 53.7 | 102.1 |
|  | ASV | n.d | n.d. |
|  | ASV (chromosomal) | 79.1 | 206.7 |
| eGFP | LVA | 49.0 | 74.2 |
|  | AAV | 113.4 | 170.2 |
|  | ASV | n.d | n.d. |
|  | ASV (chromosomal) | 67.1 | 134.3 |
| mNeonGreen | LVA | 5.9 | 11.0 |
|  | AAV | 10.5 | 18.0 |
|  | ASV | 105.2 | n.d. |
|  | ASV (chromosomal) | 30.6 | 55.6 |
| mScarlet | LVA | 166.4 | n.d. |
|  | AAV | n.d | n.d |
|  | ASV | n.d | n.d |
|  | ASV (chromosomal) | 176.0 | 220.2 |


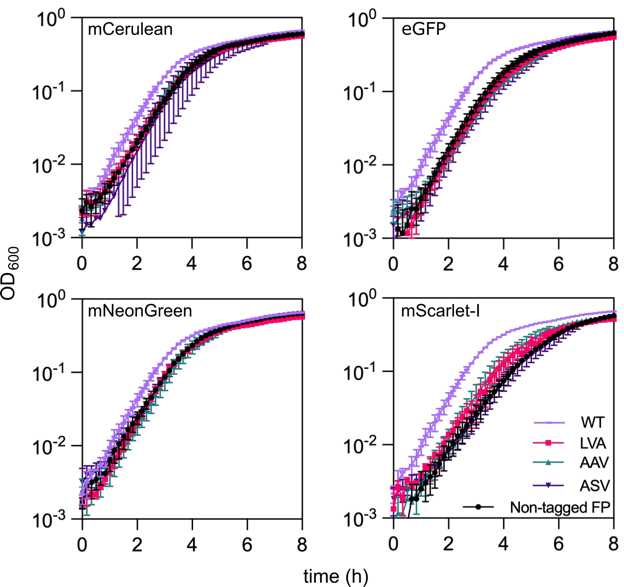


**Figure S2 Growth of *S*. Typhimurium harboring plasmids for expression of FPs fused to SsrA-tags**

Wild-type (WT) *S*. Typhimurium and strains expressing indicated FPs either non-tagged or fused to one of the three SsrA degradation tags. Optical density (OD_600_) was measured every 10 min over a period of eight hours. Depicted means and standard deviation derive from three biological independent experiments


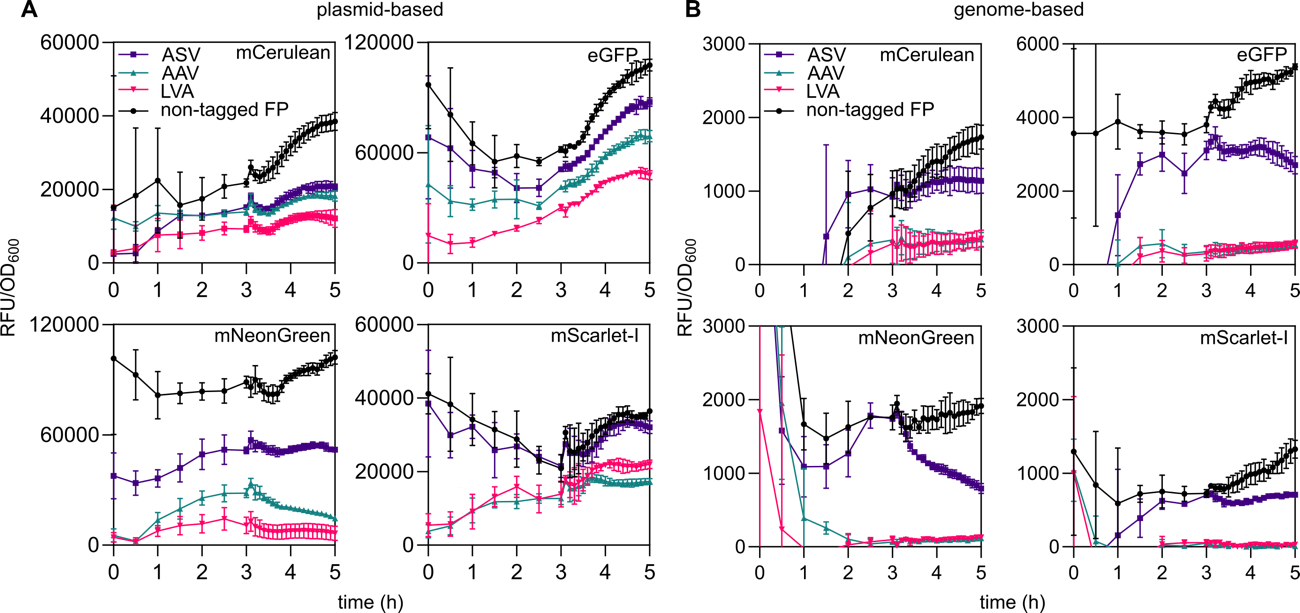


**Figure S3: Plasmid and chromosomal FPs fused to SsrA degradation tags**

Strains expressing indicated FPs either non-tagged or fused to one of the three SsrA tags. Fluorescence expression was monitored over a period of 5h and normalized to the optical density (OD_600_). Depicted means and standard deviation derive of at least two biological independent experiments.


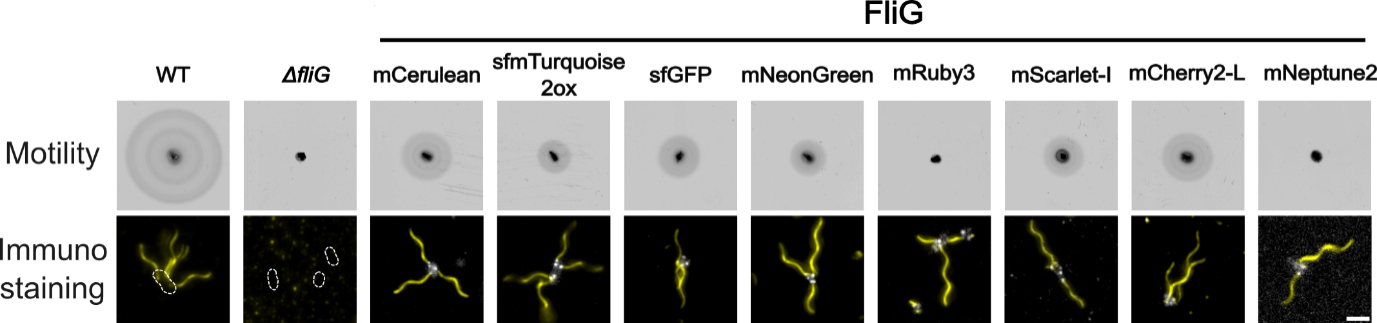


**Figure S4: Observation of swimming motility and flagellation in wild-type, Δ*fliG* and FliG N-terminal translational fusion.** Top panel: Motility assay results depicting the swimming capabilities of wild-type (WT), Δ*fliG* and FliG FPs N-terminal translational fusion strains. The motility is visualized by the expansion of the bacterial growth from a central point on a semi-solid agar plate. Bottom panel: Immunostaining images showing the flagellar filament of the strains indicated above after immunostaining of the flagellin. The images highlight the localization and distribution of the flagellar filament. Scale bar = 2 µm.

**Table S3: Strains used in this study**

| **Strain** | **Genotype** | **Source** |
| --- | --- | --- |
| EM7063 | LT2 / pEM2543 (pKH70-P*_rpsM_*-*eGFP*-RPAANDENYALVA) | This study |
| EM7064 | LT2 / pEM2544 (pKH70- P*_rpsM_*-*mNeonGreen*-RPAANDENYALVA) | This study |
| EM7065 | LT2 / pEM2545 (pKH70- P*_rpsM_*-*mCerulean*-RPAANDENYALVA) | This study |
| EM7066 | LT2 / pEM2546 (pKH70- P*_rpsM_*-*mScarlet*-*I*-RPAANDENYALVA) | This study |
| EM7067 | LT2 / pEM7067 (pKH70- P*_rpsM_*-*eGFP*-RPAANDENYAAAV) | This study |
| EM7068 | LT2 / pEM7068 (pKH70- P*_rpsM_*-*eGFP*-RPAANDENYAASV) | This study |
| EM7069 | LT2 / pEM7069 (pKH70- P*_rpsM_*-*mScarlet-I*-RPAANDENYAAAV) | This study |
| EM7070 | LT2 / pEM7070 (pKH70- P*_rpsM_*-*mNeonGreen*-RPAANDENYAASV) | This study |
| EM7071 | LT2 / pEM7071 (pKH70- P*_rpsM_*-*mScarlet*-*I*-RPAANDENYAASV) | This study |
| EM7072 | LT2 / pEM7072 (pKH70- P*_rpsM_*-*mNeonGreen*-RPAANDENYAAAV) | This study |
| EM7073 | LT2 / pEM7073 (pKH70- P*_rpsM_*-*mCerulean*-RPAANDENYAAAV) | This study |
| EM7074 | LT2 / pEM7074 (pKH70- P*_rpsM_*-*mCerulean*-RPAANDENYAASV) | This study |
| EM8313 | pEM8313 (pKH70-P*_rpsM_*-*mCerulean*, AmpR) | This study |
| EM8313 | pEM8318 (pKH70-P*_rpsM_*-*sfmTurquoise2ox*, AmpR) | This study |
| EM8314 | pEM8314 (pKH70-P*_rpsM_*-*mRuby3*, AmpR) | This study |
| EM8315 | pEM8315 (pKH70-P*_rpsM_*-*mCherry2-L*, AmpR) | This study |
| EM8317 | pEM8317 (pKH70-P*_rpsM_*-*sfGFP*, AmpR) | This study |
| EM8731 | pEM8731 (pKH70-P*_rpsM_*-*mNeonGreen*, AmpR) | This study |
| EM8733 | pEM8733 (pKH70-P*_rpsM_*-*mCherry*, AmpR) | This study |
| EM8734 | pEM8734 (pKH70-P*_rpsM_*-*Ypet*, AmpR) | This study |
| EM9177 | pEM9177 (pKH70-P*_rpsM_*-*mGFPmut2*, AmpR) | This study |
| EM10686 | pEM10686 (pKH70 P*_rpsM_*-*mScarlet*-*I*, AmpR) | This study |
| EM10687 | pEM10687 (pKH70-P*_rpsM_*-*mNeptune2*, AmpR) | This study |
| EM10688 | *pEM10688 (pKH70-P_rpsM_-mNeptune2.5, AmpR)* | This study |
| EM10689 | pEM10689 (pKH70-P*_rpsM_*-*miniSOG*, AmpR) | This study |
| EM10694 | pEM10694 (pKH70-P*_rpsM_*-*mKelly2*, AmpR) | This study |
| EM11737 | Δ*amyA::*KanSceI *(*ΔaaM1*-*Stopaa495) / pWRG730 | This study |
| EM12395 | pEM12395 (pKH70-P*_rpsM_*-*mVenusNB*, AmpR) | This study |
| EM13792 | Δ*amyA*::P*_rpsM_*-*eGFP* / pWRG730 | This study |
| EM13793 | Δ*amyA*::P*_rpsM_*-*mNeonGreen* / pWRG730 | This study |
| EM13794 | Δ*amyA*::P*_rpsM_*-*mCerulean* / pWRG730 | This study |
| EM13805 | Δ*amyA*::P*_rpsM_*-*eGFP* | This study |
| EM13806 | Δ*amyA*::P*_rpsM_*-*mNeonGreen* | This study |
| EM13807 | Δ*amyA*::P*_rpsM_*-*mCerulean* | This study |
| EM13813 | Δ*amyA*::P*_rpsM_*-*eGFP*::*KanSceI* (KanSceI replacing stop codon of *eGFP*) / pWRG730 | This study |
| EM13814 | Δ*amyA*::P*_rpsM_*-*mNeonGreen*::KanSceI (KanSceI replacing stop codon of *mNeongreen*) / pWRG730 | This study |
| EM13815 | Δ*amyA*::P*_rpsM_*-*mCerulean*::KanSceI (KanSceI replacing stop codon of *mCerulean*) / pWRG730 | This study |
| EM13816 | Δ*amyA*::P*_rpsM_*-*mScarlet*-I::KanSceI (KanSceI replacing stop codon of *mScarlet*-I) / pWRG730 | This study |
| EM13825 | pEM13825 (pKH70-P*_rpsM_*-*eGFP*) | This study |
| EM13844 | Δ*amyA*::P*_rpsM_*-*eGFP*-RPAANDENYALVA | This study |
| EM13845 | Δ*amyA*::P*_rpsM_*-*eGFP*-RPAANDENYAAAV | This study |
| EM13846 | Δ*amyA*::P*_rpsM_*-*eGFP*-RPAANDENYAASV | This study |
| EM13847 | Δ*amyA*::P*_rpsM_*-*eGFP*-AANDENYALVA | This study |
| EM13848 | Δ*amyA*::P*_rpsM_*-*mNeonGreen*-RPAANDENYALVA | This study |
| EM13849 | Δ*amyA*::P*_rpsM_*-*mNeonGreen*-RPAANDENYAAAV | This study |
| EM13850 | Δ*amyA*::P*_rpsM_*-*mNeonGreen*-RPAANDENYAASV | This study |
| EM13851 | Δ*amyA*::P*_rpsM_*-*mNeonGreen*-AANDENYALVA | This study |
| EM13852 | Δ*amyA*::P*_rpsM_*-*mCerulean*-RPAANDENYALVA | This study |
| EM13853 | Δ*amyA*::P*_rpsM_*-*mCerulean*-RPAANDENYAAAV | This study |
| EM13854 | Δ*amyA*::P*_rpsM_*-*mCerulean*-RPAANDENYAASV | This study |
| EM13855 | Δ*amyA*::P*_rpsM_*-*mCerulean*-AANDENYALVA | This study |
| EM13856 | Δ*amyA*::P*_rpsM_*-*mScarlet*-I-RPAANDENYALVA | This study |
| EM13857 | Δ*amyA*::P*_rpsM_*-*mScarlet*-I-RPAANDENYAAAV | This study |
| EM13858 | Δ*amyA*::P*_rpsM_*-*mScarlet*-I-RPAANDENYAASV | This study |
| EM13859 | Δ*amyA*::P*_rpsM_*-*mScarlet*-I-AANDENYALVA | This study |
| EM13882 | Δ*amyA*::P*_rpsM_*-*mScarlet*-I | This study |
| EM14032 | Δ*amyA*:: P*_rpsM_*-*mScarlet*-I / pWRG730 | This study |
| EM14033 | Δ*amyA*:: P*_rpsM_*-*mScarlet*-I-RPAANDENYAASV / pWRG730 | This study |
| EM14958 | ∆*clpxP*::FRT / pEM13825 (pKH70-P*_rpsM_*-*eGFP*) | This study |
| EM14959 | ∆*clpxP*::FRT / pEM2543 (pKH70-P*_rpsM_*-*eGFP*-RPAANDENYALVA) | This study |
| EM14960 | ∆*clpxP*::FRT / pEM7067 (pKH70-P*_rpsM_*-*eGFP*-RPAANDENYAAAV) | This study |
| EM14961 | ∆*clpxP*::FRT / pEM7068 (pKH70-P*_rpsM_*-*eGFP*-RPAANDENYAASV) | This study |
| EM10385 | *fliG*23319 (mCerulean-FliG N-ter, SAGASA) Δ*hin*-5717::FCF (*fliC*-ON) | This study |
| EM10386 | *fliG*23267 (sfmTurquoise2ox-FliG N-ter, SAGASA) Δ*hin*-5717::FCF (*fliC*-ON) | This study |
| EM10388 | *fliG*22798 (sfGFP-fliG) Δ*hin*-5717::FCF (*fliC*-ON) | This study |
| EM10391 | *fliG*22799 (mNeonGreen-FliG) Δ*hin*-5717::FCF (*fliC*-ON) | This study |
| EM10392 | *fliG*23318 (mRuby3-FliG N-ter, SAGASA) Δ*hin*-5717::FCF (*fliC*-ON) | This study |
| EM10577 | *fliG*23343 (mScarlet-I-FliG N-ter, SAGASA) Δ*hin*-5717::FCF (*fliC*-ON) | This study |
| EM10394 | *fliG*23324 (mCherry2-L-FliG N-ter, SAGASA) Δ*hin*-5717::FCF (*fliC*-ON) | This study |
| EM10395 | *fliG*23281 (mNeptune2-FliG N-ter, SAGASA) Δ*hin*-5717::FCF (*fliC*-ON) | This study |
| EM10568 | Δ*fliG*6012::FRT Δ*hin*-5717::FCF (*fliC*-ON) | This study |
| TH5861 | Δ*hin*-5717::FCF (*fliC*-ON) | Lab collection |
| TH437 | *Salmonella enterica* serovar Typhimurium LT2 | J. Roth |

**Table S4: Oligonucleotides used in this study**

| **Primer** | **Name** | **Sequence (5‘-3‘)** |
| --- | --- | --- |
| 3185 | 5'-EcoRI-*mCerulean*-fw | ggacagaattcataaggaggaaaaacatATGGTTAGTAAAGGGGAG |
| 3186 | 3'-NotI-*mCerulean*-rev | ATGCCTCTAGAGCGGCC |
| 3187 | 5'-EcoRI-*mRuby3*-fw | ggacagaattcataaggaggaaaaacatATGGTGTCTAAGGGCGA |
| 3188 | 3'-NotI-*mRuby3*-rev | gtactgcggccgcTTACTTGTACAGCTCGTCCA |
| 3189 | 5'-EcoRI-*sfmTurquoise2ox* | ggacagaattcataaggaggaaaaacatatgGTGAGCAAGGGCGAGGA |
| 3190 | 3'-NotI-*sfmTurquoise2ox* | gtactgcggccgcTTATCACTTGTACAGCTCGT |
| 3191 | 5'-EcoRI-*mCherry2*-L-fw | ggacagaattcataaggaggaaaaacatATGGTCTCTAAAGGCGAG |
| 3192 | 3'-NotI-*mCherry2*-L-rev | gtactgcggccgcTTATCATTTATATAACTCGTCC |
| 3193 | 5'-EcoRI-*sfGFP*-fw | ggacagaattcataaggaggaaaaacatatgTCTAAAGGTGAAGAACTGTT |
| 3194 | 3'-NotI-*sfGFP*-rev | gtactgcggccgcTTATCATTTGTAGAGCTCAT |
| 3195 | 5'-EcoRI-*mNeonGreen*-fw | ggacagaattcataaggaggaaaaacatATGGTATCGAAGGGCGAG |
| 3196 | 3'-NotI-*mNeonGreen*-rev | gtactgcggccgcttaTCATTTATACAGTTCATCCATGCC |
| 3300 | 5'-EcoRI-*mCherry*-cloning-fw | ggacagaattcataaggaggaaaaacatATGGTTTCCAAGGGCGAGGA |
| 3301 | 3'-NotI-*mCherry*-cloning-rev | CCTTTCGTTTTATTTGATGC |
| 3302 | 5'-EcoRI-pYPet-cloning-fw | ggacagaattcataaggaggaaaaacatATGTCTAAAGGTGAAGAATT |
| 3561 | 3'-NotI-pYPet-STOP-cloning-rev | gtactgcggccgcttatcaTTTGTACAATTCATTCATAC |
| 3562 | 5'-P*^rpsM^*-*mGFPmut2*-BamHI-fw | accgcggatccCAGATGGAGTTCTGAGGTCA |
| 3563 | 3'-*mGFPmut2*-NotI-rev | gtactgcggccgcttaTTATCATTTATATAACTCAT |
| 4058 | 5'-*mScarlet*-I-EcoRI-fw | ggacagaattcataaggaggaaaaacatATGGTGAGCAAAGGCGAAGC |
| 4059 | 3'-*mScarlet*-I-XbaI-rev | ctgagctctagattattaTTTATACAGTTCATCCATGC |
| 4059 | 3'-*mScarlet*-I-XbaI-rev | ctgagctctagattattaTTTATACAGTTCATCCATGC |
| 4060 | 5'-*miniSOG*-EcoRI-fw | ggacagaattcataaggaggaaaaacatATGGAAAAAAGCTTTGTGA |
| 4061 | 3'-*miniSOG*-NotI-rev | gtactgcggccgctcattaGCCATCCAGCTGCACGCCA |
| 4064 | 5'-*mKelly2*-EcoRI-fw | ggacagaattcataaggaggaaaaacatATGGAACTGATTAAAGAAA |
| 4065 | 3'-*mKelly2*-NotI-rev | gtactgcggccgctcattaGCGCGCCACCGCCACTTCA |
| 4066 | 5'-*mNeptune*-EcoRI-fw | ggacagaattcataaggaggaaaaacatATGGTGAGCAAAGGCGAAG |
| 4067 | 3'-*mNeptune*-NotI-rev | gtactgcggccgctcattaTTTATACAGTTCATCCATGC |
| 4797 | 5'-*mVenusNB*-EcoRI | ggacagaattcataaggaggaaaaacatATGAGCAAAGGCGAAGAACT |
| 6349 | 3‘-*amyA*-RPAANDENYAlva_rv | atttcgcttcccggcagcgctctgccgccgggaacgctcaTTATTAAGCTACTAAAGCG |
| 6350 | 5‘-*mScarlet*_RPAANDENYAlva_fw | gaaggccgccatagcaccggcggcatggatgaactgtataaaAGGCCTGCTGCAAACGAC |
| 6351 | 5‘-*mNeongreen*_RPAANDENYAlva_fw | aaagcatttaccgatgtaatgggcatggatgaactgtataaaAGGCCTGCTGCAAACGAC |
| 6352 | 5‘-*mCerulean*_RPAANDENYAlva_fw | ggcggccgggattactttggggatggacgagttatataagAGGCCTGCTGCAAACGAC |
| 6353 | 5‘-*eGFP*_RPAANDENYAlva_fw | accgccgccgggatcactctcggcatggatgaactgtataaaAGGCCTGCTGCAAACGAC |
| 6354 | 5‘-RPAANDENYAaav | AGGCCTGCAGCAAACGACGAAAACTACGCTGCAGCAGTTTAATAA |
| 6355 | 3‘-*amyA*-RPAANDENYAaav_rv | atttcgcttcccggcagcgctctgccgccgggaacgctcaTTATTAAACTGCTGCAGCG |
| 6356 | 5‘-RPAANDENYAasv | AGGCCTGCAGCAAACGACGAAAACTACGCTGCATCAGTTTAATAA |
| 6357 | 3‘-*amyA*-RPAANDENYAasv_rv | atttcgcttcccggcagcgctctgccgccgggaacgctcaTTATTAAACTGATGCAGCG |
| 6358 | 5‘-*mScarlet*_RPAANDENYAaav/asv_fw | aaggccgccatagcaccggcggcatggatgaactgtataaaAGGCCTGCAGCAAACGACG |
| 6359 | 5’-*mNeongreen*RPAANDENYAaav/asv_fw | aagcatttaccgatgtaatgggcatggatgaactgtataaaAGGCCTGCAGCAAACGACG |
| 6360 | 5’-*mCerulean*RPAANDENYAaav/asv_fw | ggcggccgggattactttggggatggacgagttatataagAGGCCTGCAGCAAACGACG |
| 6361 | 5‘-*eGFP*_RPAANDENYAaav/asv_fw | ccgccgccgggatcactctcggcatggatgaactgtataaaAGGCCTGCAGCAAACGACG |
| 6362 | 3‘-*amyA*-AANDENYAlva_rv | atttcgcttcccggcagcgctctgccgccgggaacgctcaTTATCACGCCACCAGCGCAT |
| 6363 | 5‘-*mScarlet*_AANDENYALVA_fw | gaaggccgccatagcaccggcggcatggatgaactgtataaaGCGGCGAACGATGAAAAC |
| 6364 | 5‘-*eGFP*_AANDENYALVA_fw | accgccgccgggatcactctcggcatggatgaactgtataaaGCGGCGAACGATGAAAAC |
| 6365 | 5‘-*mNeongreen*_AANDENYALVA_fw | aaagcatttaccgatgtaatgggcatggatgaactgtataaaGCGGCGAACGATGAAAAC |
| 6366 | 5‘-*mCerulean*_AANDENYALVA_fw | ggcggccgggattactttggggatggacgagttatataagGCGGCGAACGATGAAAAC |
| 6414 | 3'-XbaI-RPAANDENYAlva_rv | tttgatgcctctagaTTATTAAGCTACTAAAGCG |
| 6415 | 3'-XbaI-RPAANDENYAaav_rv | tttgatgcctctagaTTATTAAACTGCTGCAGCG |
| 6416 | 3'-XbaI-RPAANDENYAasv_rv | tttgatgcctctagaTTATTAAACTGATGCAGCG |
| 6417 | 3'-XbaI-AANDENYAlva_rv | tttgatgcctctagaTTATCACGCCACCAGCGC |
